# The Effect of Social Networking Site Use on Depression in Graduate Students: The Mediating Role of Negative Social Comparison and Moderating Role of Implicit Personality Theories

**DOI:** 10.3390/bs13050412

**Published:** 2023-05-15

**Authors:** Zhenzhen Chen, Yang Wu, Hongyu Ma, Gengfeng Niu, Weixin Wang

**Affiliations:** 1 School of Psychology, Central China Normal University, Wuhan 430079, China; 2 Students’ Mental Health Center, Central China Normal University, Wuhan 430079, China; 3 School of Marxism, Huazhong University of Science and Technology, Wuhan 430074, China

**Keywords:** social networking site, negative social comparison, depression, graduate students

## Abstract

Objective: The current study aimed to investigate the effect of SNS use on graduate students’ depression and further explored the effect of negative social comparison and an individual’s implicit personality theory. Methods: Scales for Social Networking Site Use Intensity, the Negative Social Comparison Measure, the Implicit Personality Theory Inventory, and CES-D were used to investigate 1792 graduate students from a full-time university in Wuhan. Result: (1) Social networking site use was positively correlated with negative social comparison and depression; (2) the mediating effect of negative social comparison was significant in social networking site use’s influence on depression; (3) after controlling for negative social comparison, graduate students’ use of SNS could negatively predict depression; and (4) the mediation effect of negative social comparison was moderated by an individual’s implicit personality theory. Specifically, the mediation effect was more pronounced among the entity theorists, while the graduate students’ incremental implicit personality theory may buffer the depressive effect of negative social comparison. Conclusions: Negative social comparison mediates the relationship between SNS use and depression; in addition, individual differences in implicit personality theory (the entity theorist vs. incremental theorist) moderate the link between negative social comparison and depression.

## 1. Introduction

In recent years, mental health problems among graduate students have become increasingly noticeable and have received considerable social attention. Graduate students generally face multiple external pressures, ranging from academic, economic, and social adjustment to work–family conflicts, which may have contributed to a much higher positive rate of depression among this population. One study found that 39% of graduate students have reported moderate to severe depressive symptoms, a rate far higher than that (6%) of the general population [1]. Another study also showed that 32% of Ph.D. candidates have a heightened risk for mental disorders, especially depression; the ratio is significantly higher among groups who have received higher education and college students [2]. A typical protective factor against mental health problems for any individual, including graduate students, is good interpersonal relationships, whereby individuals could gain a buffer against stress, regain a sense of control, replenish coping resources, and thus keep themselves mentally healthy [3]. In the current digital era, various interpersonal relationships of graduate students, including mentorship, peer support, family support, and other social support [4], are increasingly operating and being maintained via social media platforms (especially social networking sites, SNS) with greater efficiency and immediacy [5]. Given their pervasiveness and easy accessibility, social media platforms may help promote greater online self-disclosure [6] and develop a sense of belongingness in online communities [7], thus increasing one’s social connectedness and social capital [8], which are all beneficial to mental health. Therefore, interpersonal interactions on social media serve an important coping function for graduate students in combating stresses stemming from loneliness, overwork, and competition.

However, a growing body of research has identified a series of downsides to SNS usage. In those studies, SNS usage was usually indexed by use time, rate, level of immersion, or addiction. In a seminal work, researchers [9] formulated a more inclusive notion of SNS use intensity by combining the indices of time, number of friends, emotional involvement, daily life involvement, etc. Following this operationalization, studies on adolescents and adults both found that intense SNS usage may have deleterious effects on mental health [10,11,12], and as their social media use intensity increases, users tend to report more depressive symptoms [13]. Several meta-analyses also revealed that SNS usage is positively correlated with depression (*r* = 0.14–0.16) across studies [14,15]. This could be due to the superficial nature of most interpersonal interactions on social media platforms and other smartphone-based apps, which may have limited the scope and depth of online self-disclosure and is thus unable to bring about the deep social connectedness and intimate experiences that people really need [6]. Consistent with this, a study showed that the kind of social support that is the most available on social media is not correlated with mental health [16]. Based on this, the present study hypothesizes that graduate students’ use of SNS is positively associated with their depressive symptoms (H1); specifically, the higher the SNS use intensity, the higher the levels of the graduate students’ depressive symptoms. 

Nevertheless, it is also notable that the relationship between SNS usage and mental health is complex and is subject to the influence of how the SNS is used and individual differences. The present study also aims to explore the possible role of negative social comparison in the link between SNS usage and depression, as well as the possible moderating role of implicit theories of personality in the link between SNS usage and depression. 

### 1.1. Mediating Role of Negative Social Comparison

Whether the SNS-based social interaction is positive or negative is key to understanding its impacts on individual mental health [17]. Research has shown that SNS usage could be beneficial to mental health if users could build meaningful interpersonal ties through its use [17,18], whereas it would be harmful to self-esteem or well-being if they were inundated by negative interpersonal feedbacks [19]. A study on undergraduate students found that it was the quality of the experience of SNS-based interpersonal interaction, but not the mere rate of usage, that predicted depression [20]. Furthermore, a recent study showed that the negative feelings from social comparisons on social media are more damaging to one’s self-evaluation than those elicited in other contexts [21]. Based on this, the present study aims to explore the role of negative social comparison, a ubiquitous and inevitable phenomenon during SNS usage. 

Negative social comparison refers to the negative feelings resulting from social comparisons during social media use. It focuses on the emotional outcome of the comparison rather than the direction (e.g., upward vs. downward) or frequency. Negative social comparison generally stems from overexposure to positively biased content feeds on social media, which engender negative self-evaluation (e.g., thoughts such as “I am worse than others”) and unpleasant emotional feelings [22]. Social networking sites are important venues for self-presentation [23], in which individuals under self-enhancement motives may strategically present their favored self-image and relatively pleasant experiences [24]. Consequently, social networking sites abound with positively skewed content that tends to elicit social comparison. Thus, frequent SNS users are constantly exposed to comparison cues and are more likely to fall prey to its influence. Chou and Edge [25] showed that college students spending more time using SNS were more likely to believe that other people were happier and lived better than they were. Relatedly, Frison and Eggermont [26] demonstrated that the negative social comparison occasioned by SNS usage may affect individuals’ life satisfaction; moreover, even after controlling for the role of general social comparison, negative social comparison could still predict depressive symptoms [27]. Past research has also shown that negative social comparison mediated the effect of SNS usage on depression [28]. Based on this, the present study aims to replicate past findings and hypothesizes that negative social comparison could mediate the effect of SNS usage on graduate students’ depressive symptoms (H2). 

### 1.2. Moderating Role of Implicit Theories of Personality

Numerous studies have documented several moderators in the link between SNS usage and depression, such as self-concept clarity, neuroticism, and self-esteem [21,28,29]. According to the diathesis–stress model of depression [30], stresses from the external environment are moderated by a slew of individual difference factors before influencing depression. Implicitly representing personality as an unchanging entity or a constantly evolving conglomeration may shift one’s attention to focus on stressful information and change one’s interpretation of stressful events as well as the corresponding coping strategy [31].

According to Dweck and colleagues [32,33], implicit theories of personality refer to the basic cognitive schema or naïve theories that lay persons hold about human nature. Two such belief frameworks that have received continued research attention are entity theory and incremental theory. The former posits that personality is fixed, static, and unalterable, whereas the latter posits that personality is plastic, changeable, and constantly evolving [32]. The belief differences between people holding the two theories may have bifurcating downstream effects on their attentional allocation, interpretational tendencies, and coping styles to negative events. For example, Renaud and McConnell [34] showed that one’s self-discrepancies may lower self-esteem to a greater extent for entity theorists than for incremental theorists because negative experiences would be more likely to be attributed to unalterable, dispositional causes for entity theorists, which may heighten the blow. On the other hand, incremental theorists may perceive such experiences as opportunities to better themselves, thus casting the negative events in a positive light. Research did show that entity theorists tend to exhibit increased attention bias to negative events, are more likely to experience intrusive thoughts over them [31], and report more mental health conditions [35]. In contrast, the incremental mindset seems to serve as a buffer between stressful feelings and negative psychological outcomes [36], and the incremental mindset has been shown to be negatively correlated with mental health conditions such as depression [37]. Therefore, it is plausible that entity theorists, compared with incremental theorists, may be more strongly affected by negative social comparisons and develop depressive symptoms. Based on this, the present study further hypothesizes that graduate students’ implicit theories of personality may moderate the link between negative social comparison and depression, or the latter half of the mediation model (H4). Specifically, entity theorists would be more vulnerable to negative social comparison.

To sum up, the present study aims to investigate the role of SNS use on graduate students’ depression and to examine a hypothesized moderated mediation model that involves the role of negative social comparison and implicit theories of personality.

## 2. Materials and Methods

### 2.1. Participants and Procedure

A convenience sample of graduate students from a university in central China participated in the study via classroom invitation. All participants were first- or second-year full-time graduate students. After informed consent, the participants completed the questionnaire on weijuanxin.com, an online survey platform. Items from each scale were randomized, and an item for attentional check was inserted. Responses that failed the attentional check were discarded. 

Among the 1792 valid responses (valid rate: 75.4%), 937 (52.3%) were male, and 855 (47.7%) were female; the majors of the participants were engineering (903, 50.4%), science (31.5%), fine arts (13.8%), and others (77, 4.3%); 135 were Ph.D. students (7.5%), and 1657 were master students (92.5%). The average age of the participants was 23.266 (*SD* = 5.435).

### 2.2. Measurement

#### 2.2.1. Depression

The Chinese version of the Center for Epidemiologic Studies Depression Scale (CES-D Scale) was used to measure depressive symptoms. The 20-item scale adopted a Likert scale ranging from 1 (*rarely*) to 4 (*most or all of the time*), whereby participants report their symptom frequencies in the past week (e.g., my sleep was restless). In the present study, Cronbach’s α = 0.859.

#### 2.2.2. SNS Use Intensity

Adapting from Ellison, Steinfield and Lampe [9], Niu, et al. [38] developed a scale that assesses SNS use intensity. The 8-item scale consisted of 2 items that ask participants to report their number of friends on SNS and average daily use time and another 6 items that were rated on a 5-point Likert scale (1 = *Strongly disagree*–5 = *Strongly agree*) assessing their emotional connection to SNS and the extent to which SNS is embedded within daily life (e.g., SNS is part of my daily activities). As with past studies (e.g., [28]), each item was standardized before combining the items into a single index tapping participants’ SNS use intensity. The scale was shown to be reliable in the past, and in the present study, Cronbach’s α = 0.869.

#### 2.2.3. Negative Social Comparison on SNS

A brief 3-item scale used by Lee [22] was adopted to measure the extent to which participants have negative feelings from social comparisons on SNS. An example item is “When I read news feeds (or see others’ photos), I often think that others are having a better life than me”. Participants rate their agreement with the items on a 5-point Likert scale (1 = “*Strongly Disagree*” to 5 = “*Strongly Agree*”). A higher score indicates more negative feelings from social comparison during SNS use. In the present study, Cronbach’s α = 0.730.

#### 2.2.4. Implicit Theories of Personality

The scale used in measuring participants’ implicit theories of personality was developed by Chiu, Hong and Dweck [33]. The scale consisted of 3 items, for example, “People can do things differently, but the important parts of who they are can’t really be changed.” Participants rate their agreement with the items on a 7-point Likert scale (1 = “*Strongly Disagree*” to 7 = “*Strongly Agree*”), with a higher score indicating a tendency towards the entity theory of personality and a lower score indicating a tendency towards the incremental theory of personality. In the present study, Cronbach’s α = 0.762.

### 2.3. Statistical Analysis

Data were prepared and analyzed by using IBM SPSS 22.0. The moderated mediation model was tested by using the SPSS macro PROCESS [39] model 14. The common method bias was tested using the sem package [40] in R programming language.

## 3. Results

### 3.1. Common Method Bias

The present study primarily used self-report data, which may suffer from common method bias. Following the recommendation from Podsakoff, et al. [41], several procedural precautions were implemented, such as anonymity and randomness in item order. Furthermore, we conducted Harman’s single factor test by fitting the data to a unidimensional model, and a confirmatory factor analysis found that the modeling iteration cannot converge, suggesting a bad model fit. This also indicated that no significant common method bias exists in the present study. 

### 3.2. Descriptive Statistics

The descriptive statistics (means and SDs) of the key variables and their intercorrelations are shown in Table 1. Correlational results showed that all variables were positively correlated, their coefficients ranging from 0.408 to 0.081. Critical to the hypothesis of the present study, graduate students’ use of SNS is positively associated with depression. 

### 3.3. Test of the Moderated Mediation Model

**Figure 1:**
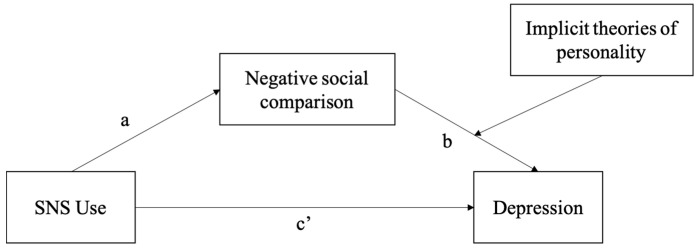
Hypothesized moderated mediation model.

To further examine the relationship between graduate students’ SNS use and depression, as well as the possible role of negative social comparison and implicit theories of personality, we tested a hypothesized moderated mediation model (Figure 1) by multiple regression as well as the SPSS macro PROCESS (model 14) with 5000 bootstrap resamples.

The results (Table 2) revealed that after controlling for gender and age, graduate students’ SNS use significantly predicted their negative social comparisons (a path), *β* = 0.388, *t* = 18.611, *p* < 0.001. Negative social comparison, in turn, could significantly predict depression (b path), *β* = 0.186, *t* =16.431, *p* < 0.001, when both direct and indirect paths were added to the model. The direct path (c’ path) from SNS use to depression was also significant but opposite in direction, *β* = −0.045, *t* = −4.220, *p* < 0.001. Given this, a separate test based on the bootstrap method showed that the mediation effect of negative social comparison (i.e., the a × b path) was significant. The opposite direction of the total effect and direct effect of SNS use on depression suggests a suppression effect of the indirect path. In other words, the present finding suggests that the increase in depression associated with greater SNS use was primarily due to negative social comparison; after teasing apart the indirect effect, graduate students’ use of SNS may help lower depressive symptoms (c’ path). 

**Figure 2:**
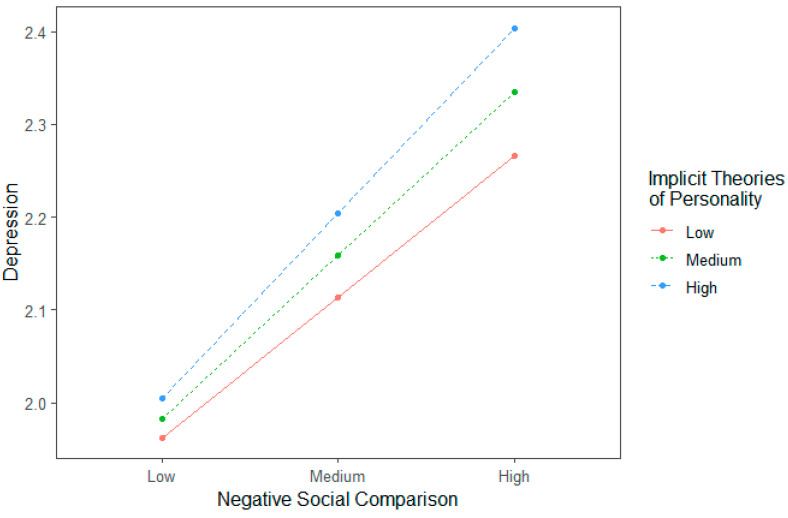
Simple slopes of the moderation effect of implicit theories of personality. Higher scores for implicit theories of personality suggest a tendency towards the entity theory, while lower scores suggest a tendency towards the incremental theory.

Furthermore, the interaction term was also significant, *β* = 0.021, *t* = 2.655, *p* = 0.008, indicating that the students’ implicit theories of personality have moderated the latter half of the mediation, i.e., the link between negative social comparison and depression. To better illustrate the moderation effect, we examined simple slopes at low, medium, and high scores of implicit theories of personality (M ± 1 SD) and treated the “high” score as indicating a tendency towards entity theory and the “low” score as indicating a tendency towards incremental theory. As shown in Table 3 and Figure 2, results from simple slope analysis showed that students with higher scores on implicit theories of personality (entity theorists) would be more likely to be affected by negative social comparison and develop depressive symptoms. In contrast, incremental theorists were relatively less affected. 

## 4. Discussion

To investigate how SNS use impacts graduate students’ depression and its mechanism, the present study formulated a moderated mediation model. The results showed that graduate students’ SNS use is positively correlated with depression, and the link is mediated by negative social comparison. Graduate students’ implicit theories of personality moderated the latter half of the mediation model; specifically, entity theorists tended to be more affected by the negative social comparison occasioned by SNS use to develop depressive symptoms. 

### 4.1. Relationship between SNS Use Intensity and Depression

The present results found that, holding constant other factors and boundary conditions, the greater use of SNS has generally ameliorative effects on depressive symptoms among graduate students; the correlation is weak but significant. This is inconsistent with results from most other populations [12,14].

However, in past studies, evidence regarding the potential impacts of SNS use on mental health has been mixed. On the one hand, there is evidence showing that the positive use of SNS may help obtain social support [42] and elevate users’ life satisfaction and social adaptability. On the other hand, the negative use of SNS has also been shown to have a harmful influence on mental health, such as increased depressive symptoms [27]. The mixed findings suggest the existence of potential mediating paths that are parallel yet have opposite valences, which may be subject to certain boundary conditions. 

In line with this, the present study examined the mediating role of negative social comparison and found that, consistent with H2, the effect of SNS on depression is indeed mediated by students’ negative social comparison on SNS. Typically, an individual’s propensity to strategic online self-presentation often saturates SNS feeds with positively biased content only highlighting the brighter side of daily life [23]. Since people spend most of their time on SNS browsing through content feeds from friends [43,44], they are constantly exposed to the biased content, triggering negative social comparisons that culminate in negative emotional experiences and negative self-evaluation. This also dovetails with social rank theory, which posits that a main source of depression is the perception that the self is inferior to others [45]. A series of related studies have demonstrated that SNS use could induce negative self-evaluation through negative social comparison, thus harming a person’s psychological well-being [27,28,46]. The present findings replicated the mediating effect of negative social comparison, lending further support to the view that the outcomes of SNS use depend on the specific ways in which it is used [17,18].

A notable finding from the present study is that once negative social comparison is controlled, the direct path between SNS use and graduate students’ depressive symptoms became negative. This finding suggests that SNS use may have ameliorative, though weak, effects on graduate students if they could abstain from negative social comparison. According to the interpersonal–connection–behaviors framework [17], SNS may promote psychological health by helping individuals gain meaningful social connections (social support, belongingness, offline connections, etc.). That is, graduate students may have partially fulfilled their need for belongingness, maintained their existing social connections, and obtained social support via SNS [47], which could all help prevent or reduce depression, even if they were simultaneously engaged in negative social comparison. In addition, graduate students may have been more intentional in their SNS use than younger population groups, such as using SNS to acquire study information [48], promote offline social connection [5], cultivate bridging social capitals in academia [49], etc. Passive usage of SNS is more likely to cause anxiety [43], and there is also experimental evidence showing that the deleterious consequences of SNS use stem from random browsing rather than intentional usage [50]. Thus, though preliminary, data from the present study suggest that there could be a possibility that the relatively intentional and instrumental use of SNS among graduate students may have beneficial effects on reducing depressive symptoms. Future studies could examine this possibility by testing the mediation of this depression-ameliorative mechanism of SNS use.

### 4.2. Moderating Role of Implicit Theories of Personality

The present study also examined the possible boundary condition for the mediation model, and the results showed that graduate students’ implicit theories of personality moderated the latter half of the mediation—i.e., from negative social comparison to depression. Specifically, graduate students holding the entity theory tended to be more affected by the negative social comparison occasioned by SNS use to develop depressive symptoms. This is consistent with our hypothesis. People holding different implicit theories of personality would differ in their perception and interpretation of stressful events and other failure-related information, leading to disparate coping strategies [31]. Research on college students found that entity theorists usually reported more severe psychological conditions, whereas incremental theorists reported fewer; the coping strategies of the latter were also more positive, i.e., adopting the cognitive reinterpretation strategy more often, preferring counseling over an overreliance on drugs, etc. [37,51]. Entity theorists, in contrast, reverted to negative coping styles more regularly, which may have resulted in higher stressful reactions a year later [52]. Given this, burgeoning empirical evidence started to show that intervention programs that help cultivate and develop an incremental mindset may encourage individuals to adapt to adversities more proactively [53]. Therefore, in the present study, graduate students may have engaged themselves in negative social comparison after repeated exposure to positively biased SNS feeds; the entity theorists among them may have interpreted these negative experiences in a more dispositional manner, believing that the current state is unchangeable and adopting more negative coping styles. This exacerbates the sting of negative social comparison, leading to more depressive symptoms. Our findings from the moderated mediation model confirmed this.

### 4.3. Implications and Limitations

Findings from the present study suggest that for graduate students, the use of SNS increased depressive symptoms through negative social comparison, but after controlling for negative social comparison, SNS use could actually help reduce depression. These findings have several practical implications for the mental health of graduate students. First, graduate students should be aware of the positive bias in SNS content feeds, and thus they could intentionally avoid being embroiled in negative social comparison. Second, it is advisable that graduate students become more intentional and proactive in their use of SNS to build meaningful social connections online and harness the power of SNS for their own purpose. Last, policymakers in educational institutions could also develop intervention programs that help graduate students form a more incremental view of personality, which could serve as resilient coping resources in the event of personal setbacks or negative experiences during the use of SNS. 

There are also several limitations to the present study. First, in the present study, the cross-sectional nature of data collection precluded a causal inference of the proposed mechanism. Future studies may replicate and extend the present findings using longitudinal and experimental designs. Second, the mental health of graduate students implicates multiple factors that are not considered here. Future studies could examine the influence of other key variables, including social comparison orientation, self-esteem, the mentor–student relationship [54], and demographic factors (e.g., discipline and gender), on graduate students’ psychological well-being. Furthermore, the present study only considered an SNS platform that is devoted mostly to non-stranger social networking, and it is possible that the psychological mechanism and outcome could be different in other forms of SNS, such as TikTok, Instagram, or YouTube, on which most social interactions are stranger-to-stranger. Similarly, the psychological impacts of AI-generated chatbots (e.g., chatGPT) and their potential role in future online social interaction also deserve future research attention.

## 5. Conclusions

From a cross-sectional study on graduate students, the present study found that (1) graduate students’ SNS use intensity was positively correlated with negative social comparison and depression; (2) negative social comparison mediated the link between SNS use and depression; (3) after controlling for negative social comparison, graduate students’ use of SNS could negatively predict depression; and (4) graduate students’ implicit theories of personality moderated the link between negative social comparison and depression; specifically, the entity theorists are more affected by negative social comparison.

## Figures and Tables

**Table 1:** Descriptive statistics of key variables and their intercorrelation.

	*M* (*SD*)	1	2	3	4
1 SNS use intensity	0 (1)	1			
2 Negative social comparison	2.325 (0.944)	0.408	1		
3 Implicit theories of personality	4.314 (1.186)	0.084	0.176	1	
4 Depression	2.163 (0.443)	0.081	0.384	0.162	1

Note. All correlation coefficients were significant at 0.001 level.

**Table 2:** Moderated mediation model of SNS use’s effect on depression.

Regression Model		Model Fit			Regression Coefficients		
Criterion	Predictor	*R^*2*^*	*F*	*β*	Bootstrap Lower	Bootstrap Upper	*t*
Negative social comparison	Gender	0.167	119.601 ***	−0.030	−0.112	0.052	−0.707
	Age			−0.003	−0.010	0.004	−0.782
	SNS Use			0.388	0.347	0.429	18.611 ***
Depression	Gender	0.169	60.502 ***	0.041	0.002	0.079	2.085 *
	Age			<0.001	−0.003	0.004	0.203
	SNS Use			−0.045	−0.066	−0.024	−4.220 ***
	Negative social comparison			0.186	0.164	0.209	16.431 ***
	Implicit theories of personality			0.038	0.022	0.054	4.630 ***
	Negative social comparison × Implicit theories of personality			0.021	0.006	0.037	2.655 **

Note. Moderated mediation model tested using PROCESS macro (model 14). Index of moderated mediation is 0.008 with an SE of 0.004 and CI (0.010, 0.015) by 5000 bootstrap resamples. * *p* < 0.05, ** *p* < 0.01, *** *p* < 0.001.

**Table 3:** Mediation effect of negative social comparison at different levels of implicit theories of personality.

Implicit Theories of Personality	Mediation Effect	SE	Bootstrap Lower	Bootstrap Upper
*M* + *SD*	0.082	0.008	0.067	0.098
*M*	0.072	0.007	0.060	0.085
*M* − *SD*	0.063	0.008	0.048	0.078

*Note*. Moderated mediation model tested using PROCESS macro (model 14). The simple slopes at low, medium, and high scores of implicit theories of personality corresponded to 1 SD below mean, the mean, and 1 SD above mean. The higher the score of implicit theories of personality, the stronger the tendency towards “entity theory”.

## Data Availability

Data will be stored in a publicly accessible repository and will be available upon publication from the osf.io database (osf.io/ef6r7).
